# 肺癌肺叶切除患者术前存在气道定植菌与术后肺炎的发生有相关性吗？

**DOI:** 10.3779/j.issn.1009-3419.2017.04.03

**Published:** 2017-04-20

**Authors:** 珂 高, 玉田 赖, 健 黄, 一帆 王, 晓玮 王, 国卫 车

**Affiliations:** 1 610017 成都，成都市第二人民医院胸心外科 Department of Thoracic and Cardiovascular Surgery, the Second People's Hospital of Chengdu, Chengdu 610017, China; 2 610041 成都，四川大学华西医院胸外科 Department of Thoracic and Cardiovascular Surgery, West China Hospital of Sichuan University, Chengdu 610041, China

**Keywords:** 肺肿瘤, 致病性气道定植菌, 肺表面活性蛋白D, 术后肺炎, Lung neoplasms, Pathogenic airway bacterial colonization, Surfactant protein D (SP-D), Postoperative pneumonia (POP)

## Abstract

**背景与目的:**

外科手术是目前治疗肺癌的主要手段，肺癌患者围术期死亡的主要原因仍是术后肺炎。已有的研究结果显示致病性气道定植菌被认为是术后肺部并发症的一个独立危险因素，本研究旨在探讨术前致病性气道定植菌的存在与术后发生肺炎的关系及其危险因素。

**方法:**

横断面调查2014年5月至2015年1月连续收治于成都市6家三级甲等医院胸外科行手术治疗的125例非小细胞肺癌患者，术前经纤维支气管镜取气管及支气管内液细菌学标本，并检测术前血清肺表面活性蛋白D（surfactant protein D, SP-D）水平，术后肺部相关并发症进行分析。

**结果:**

肺癌患者术前合并致病性气道定植菌的发生率为15.2%（19/125），以革兰氏阴性菌为主（19/22, 86.36%）；肺癌患者术前合并致病性气道定植菌的高危因素为：高龄（≥75岁）和长期吸烟史（吸烟指数≥400支/年）；术后肺部相关并发症和术后肺炎发生率在肺癌合并致病性气道定植菌组（42.11%, 26.32%）均显著高于非合并组（16.04%, 6.60%）（*P*=0.021, *P*=0.019）。术前血清SP-D浓度在肺癌合并致病性气道定植菌（31.25±6.09）显著高于非合并组（28.17±5.23）（*P*=0.023）。并发术后肺炎患者中气道致病性定值菌发生率为41.67%（5/12），其发生率是无手术后肺炎患者的3.4倍（OR=3.363, 95%CI: 1.467-7.711）。

**结论:**

肺癌患者合并致病性气道定植菌与术后肺炎发生密切相关，且高危险因素是高龄和长期吸烟史。

## Abstract (en)

**Background and objective:**

Surgical procedure is the main method of treating lung cancer. Meanwhile, postoperative pneumonia (POP) is the major cause of perioperative mortality in lung cancer surgery. The preoperative pathogenic airway bacterial colonization is an independent risk factor causing postoperative pulmonary complications (PPC). This cross-sectional study aimed to explore the relationship between preoperative pathogenic airway bacterial colonization and POP in lung cancer and to identify the high-risk factors of preoperative pathogenic airway bacterial colonization.

**Methods:**

A total of 125 patients with non-small cell lung cancer (NSCLC) underwent thoracic surgery in six hospitals of Chengdu between May 2015 and January 2016. Preoperative pathogenic airway bacterial colonization was detected in all patients via fiber bronchoscopy. Patients' PPC, high-risk factors, clinical characteristics, and the serum surfactant protein D (SP-D) level were also analyzed.

**Results:**

The incidence of preoperative pathogenic airway bacterial colonization among NSCLC patients was 15.2% (19/125). Up to 22 strains were identified in the colonization positive group, with Gram-negative bacteria being dominant (86.36%, 19/22). High-risk factors of pathogenic airway bacterial colonization were age (≥75 yr) and smoking index (≥400 cigarettes/year). PPC incidence was significantly higher in the colonization-positive group (42.11%, 8/19) than that in the colonization-negative group (16.04%, 17/106)(*P*=0.021). POP incidence was significantly higher in the colonization-positive group (26.32%, 5/19) than that in the colonization-negative group (6.60%, 7/106)(*P*=0.019). The serum SP-D level of patients in the colonization-positive group was remarkably higher than that in the colonization-negative group [(31.25±6.09) *vs* (28.17±5.23)](*P*=0.023). The incidence of preoperative pathogenic airway bacterial colonization among NSCLC patients with POP was 41.67% (5/12). This value was 3.4 times higher than that among the patients without POP (OR=3.363, 95%CI: 1.467-7.711).

**Conclusion:**

An intimate correlation was observed between POP and pathogenic airway bacterial colonization in lung cancer. The high-risk factors of pathogenic airway bacterial colonization were age and smoking index.

肺癌是全球男性和发达国家女性因癌症死亡率最高的疾病种类^[1, 2]^。外科手术仍是目前治疗肺癌的主要手段，肺癌手术后肺部并发症（postoperative pulmonary complication, PPC）是影响患者手术后早期恢复的主要原因，其中手术后肺炎（postoperative pneumonia, POP）是肺癌患者围手术期主要致死原因^[3]^。致病性气道定植菌被认为是PPC的一个独立危险因素^[4]^，而肺表面活性蛋白D（surfactant protein D, SP-D）与肺内的天然免疫有关，肺内的局部免疫状态对致病性细菌定植有重要影响。为探讨肺癌患者术前致病性气道定植菌分布、血清SP-D浓度与肺叶切除术后肺炎发生的关系，我们研究了成都市6家三甲医院连续收治的125例可手术的肺癌患者，分析肺癌患者手术前气道致病性定植菌的危险因素，血清SP-D水平变化与术后肺炎发生的相关性。

## 1 资料与方法

### 1.1 临床资料

选取2014年5月-2015年1月的连续收治的四川省肿瘤医院、四川大学华西医院、成都市第二人民医院、成都市第三人民医院、成都大学附属医院、成都医学院附属第一医院等6家三级甲等医院胸外科收治的拟手术治疗的非小细胞肺癌患者192例。纳入标准：①年龄大于18岁且小于80岁；②符合四川大学华西医院生物伦理委员会批准的受试者要求，同意术前根据实验要求进行检查，并签署知情同意书；③全胸腔镜/开胸肺叶切除术+系统淋巴结清扫患者；④术后病理诊断原发性非小细胞肺癌。排除标准：①非非小细胞肺癌患者；②术中发现需要行全肺切除、联合肺叶切除和需要行肺动脉和支气道袖式成形肺叶切除术及亚肺叶切除；③术中出血量超过1, 000 mL和胸腔镜手术中转开胸，或者术后需要再次手术止血；④外院或门诊已行纤维支气管镜检测但未行分泌物细菌学检测；⑤实验期间拒绝继续按照实验计划进行细菌学及血清学检测；⑥根据临床症状，实验室检测和影像学资料诊断术前即合并肺部感染；⑦在标本检测时发现标本不合格的（标本污染，标本量少不足以完成所有指标检测）。具体患者筛查流程见图 1。根据排除标准共排除18例患者，其中术后病理证实非非小细胞肺癌4例，手术因素（术中大出血或亚肺叶切除）3例，实验期间拒绝继续按照实验计划进行细菌学及血清学检测5例，标本不合格6例，最终进入实验患者125例，其中男性95例，女性30例，年龄43岁-76岁，平均（63.48±8.69）岁；其中鳞癌60例，腺癌63例，其他病理类型2例；其中Ⅰ期67例，Ⅱ期48例，Ⅲ期10例。

**1:**
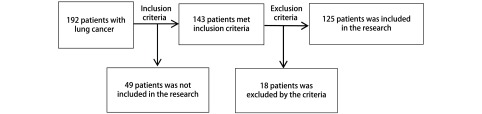
病例筛选流程图 Flow chart of case screening

### 1.2 基础肺功能（pulmonary function test, PFT）检测指标

对肺通气功能（肺容积、肺通气量、小气道功能、呼吸动力学、吸入气体分布、呼吸肌功能）和肺换气功能（弥散功能、通气血流比值）进行测定和评估。

### 1.3 肺部并发症分类及评价标准

#### 1.3.1 PPC的种类

包括：肺部感染、肺栓塞、乳糜胸、皮下气肿、咯血、声音嘶哑、支气道胸膜瘘、手术后持续肺漏气、手术后胸腔积液（中到大量）和积气（肺压缩≥30%）、肺不张、急性呼吸窘迫综合征（acute respiratory distress syndrome, ARDS）或呼吸衰竭。肺部并发症分类及评价标准见表 1。

**1:** 肺部并发症诊断标准 The criterion of postoperative pulmonary complications (PPC)

PPC	Criterion
Postoperative pneumonia	① Fever as seen by raised oral temperature of >38 ℃ in the postoperative 72 h; or temperature rise again after 72 h; ② High WBC count (open >15×10^9^, VATS >12×10^9^) or rises again over 72 h;
	③ Increasing consolidation of the lung or patchy shadows in CT/CR image(s);
	④ Positive signs of infection on sputum microbiology.
Pulmonary embolism	① Pulmonary artery angiography showing embolism;
	② Dyspnea or SpO_2_ < 90%; remission after using anticoagulant.
Chylothrax	① Raily drainage volume >500 mL, and the last 3 d or more;
	② Time of fasting≥5 d;
	③ Requiring surgical treatment.
Aerodermectasia	① Persisting >15 d, exceeding over half chest wall;
	② requiring subcutaneous incision or surgical treatment.
Hemoptysis	① Persisting >3 d and inefficacy of pharmacological treatment;
	② Requiring surgical treatment.
Voice hoarse	① Choking cough with drinking;
	② Plicavocalis paralysis or dislocation of cricoarytenoid joint.
Bronchopleural fistula	Bronchofiberscope showing bronchopleural fistula.
Air leak	① Persisting >15 d;
	② Needing interventions like re-indwelling drainage tube;
	③ Vacuum suction (>7 d), or surgical treatment.
Pleural effusion/ pneumothorax	① Drainage time >15 d, ordyspneic;
	② Requiring re-indwelling drainage tube.
Atelectasis	① Atelectasis/consolidation in CT/CR image(s);
	② Presenting dyspnea or SpO_2_ < 90% on room air.
ARDS/Respiratory failure	Needing trachea cannula and/or ventilator maintenance or intensive care.
CT: computed tomography; CR: computed radiography; ARDS: acute respiratory distress syndrome; SpO_2_: oxygen saturation.	

#### 1.3.2

术后肺炎的标准：含有以下指标3个或以上的应视为术后肺炎^[5, 6]^：①手术后72 h的发热，T>38 ℃；或72 h以内的体温再度升高。②白细胞计数升高（>12×10^9^/L-15×10^9^/L），或白细胞计数回复正常值以后的再度升高，超过10×10^9^/L。③胸部影像学提示肺组织实变或不断增加的斑片状阴影。④咳出脓性痰液，或痰培养阳性。其中如果包含④，仅需要其他一项即可视为POP。或经呼吸科会诊确定为肺部感染，并需要更换抗生素或延长抗生素使用时间。

### 1.4 气道的细菌学检查

患者在入院时，经纤维支气管镜用防污染样本毛刷或支气管肺泡灌洗液（灌洗液经过单层纱布过滤，再经过800 rpm离心10 min后，沉淀物用于微生物学检查）取细菌学标本，合格标本按标准流程预处理后，分别接种于血平板、中国蓝和巧克力平板中，在5%CO_2_、37 ℃箱体内孵化18 h后行菌落计数和分离鉴定。出现优势菌后，菌落计数。以防污染样本毛刷≥10^3^ cfu/mL、支气管肺泡灌洗液≥10^4^ cfu/mL确定为细菌定植标准^[7]^。

### 1.5 手术方法

全胸腔镜肺叶切除患者手术方法为单向式胸腔镜肺叶切除法+系统淋巴结清扫^[8]^。开胸患者应用常规后外侧切口，肺叶切除术+系统淋巴结清扫。系统淋巴结清扫左侧必须清扫第5、6、7、8、9、10组淋巴结，右侧包括第2、3、4、7、8、9、10组淋巴结。

### 1.6 术后处理方法

术后疼痛处理均应用镇痛泵（盐酸曲马多注射液，1 mg/h-1.5 mg/h），均早期促使患者下床活动。必要时应用非甾体类止痛药（氨酚羟考酮片或布洛芬缓释胶囊）。镇痛泵于胸腔引流管拔除的同时也一起停止。两组患者均根据胸腔引流气体和液体量决定行胸部X线片检查时间，并根据胸部X线片结果决定是否拔除胸腔引流管。

### 1.7 血浆中SP-D浓度的测定

在患者入院当天，采集患者外周血5 mL，待血液自然凝固10 min-20 min，离心20 min，2, 000 rpm，仔细收集上清液。应用双抗体夹心法测定标本中人SP-D水平。人SP-D ELISA试剂盒由武汉博士德生物工程有限公司提供。

### 1.8 统计学分析

使用SPSS 16.0软件进行统计分析，计量资料用独立样本的*t*检验或方差分析，计数资料用卡方检验，样本在作*t*检验和方差分析前，均先作正态分析或方差齐性检验。危险因素应用*Logistic*回归，以*P* < 0.05为差异有统计学意义。

## 2 结果

### 2.1 两组患者临床病理生理学特征

肺癌患者术前合并致病性气道定植菌19例（15.2%, 19/125），平均年龄（65.29±8.31）（46岁-76岁），78.95%（15/19）患者吸烟指数≥400支/年，68.42%（13/19）为中央型肺癌，PPC的发生率为42.11%（8/19），术后肺炎发生率为26.32%（5/19）。

106例（84.8%, 106/125）不合并致病性气道定植菌，平均年龄（62.43±7.90）（43-75）岁。吸烟指数≥400支/年的患者53.77%（57/106）。43.40%（46/106）患者为中央型肺癌，术后肺部并发症发生率16.04%（17/106），术后肺炎发生率6.60%（7/106）（表 2）。

**2:** 呼吸道气道定植菌阳性与阴性两组患者一般临床资料的比较 The clinical demographic characters of study subjects

	Postive colonization (*n*=19)	Negative colonization (*n*=106)	*χ*^2^/*t*	*P*
Gender			1.444	0.229
Male	17	78		
Female	2	28		
Age (yr)	65.29±8.31	62.43±7.90	2.107	0.032
Somking index (cigarette-year)			6.904	0.009
< 400	4	57		
≥400	15	49		
Type of pathology			0.799	0.671
AC	10	50		
SC	9	54		
Other	0	2		
Resction location			0.917	0.922
LU	3	13		
LL	6	34		
RU	6	29		
RM	1	4		
RL	3	26		
Site of tumor			4.049	0.044
Central lung cancer	13	46		
Peripheral lung cancer	6	60		
COPD level^※^			3.244	0.198
Ⅰ	2	21		
Ⅱ	14	80		
Ⅲ	3	5		
Diabetes mellitus			2.729	0.099
Yes	4	9		
No	15	97		
Hypertension			0.935	0.334
Yes	5	18		
No	14	88		
PPC			5.310	0.021
Yes	8	17		
No	11	89		
POP			5.578	0.019
Yes	5	7		
No	14	99		
AC: adenocarcinoma; SC: squamous cells carcinoma; LU: left upper lobe; LL: left lower lobe; RU: right upper lobe; RM: right middle lobe; RL: right lower lobe; COPD: chronic obstructive pulmonary disease; POP: postoperative pneumonia; PPC: postoperative pulmonary complication. ※COPD level: Ⅰ: FEV_1_≥80% (pre), FEV_1_/FVC < 70%; Ⅱ: 50% (pre)≤FEV_1_ < 80% (pre), FEV_1_/FVC < 70%; Ⅲ: 30% (pre)≤FEV_1_ < 50% (pre), FEV_1_/FVC < 70%.				

### 2.2 两组患者术后肺部并发症种类分布的比较

术后PPC的发生率在肺癌合并致病性气道定植菌组（42.11%）显著高于非合并组（16.04%）（*P*=0.021）；其中肺部感染在合并致病性气道定植菌组（26.32%）显著高于非合并组（6.60%）（*P*=0.019）（表 3）。

**3:** 两组患者术后肺部并发症种类分布的比较 The detail of PPC in colonization -/+ groups

PPC	Postive colonization (*n*=19) (8 paients, 15 PPCs)	Negative colonization (*n*=106) (17 patients, 22 PPCs)	*χ*^2^	*P*
Postoperative pneumonia	5	7	5.578	0.019
Atelectasis	3	4	3.460	0.064
Air leak	2	3	1.896	0.169
Pleural effusion/pneumothorax	2	5	0.923	0.337
Aerodermectasia	1	1	1.395	0.283
Respiratory failure	1	0	-	-
Chylothrax	0	1	-	-
Bronchopleural fistula	1	0	-	-
Voice hoarse	0	1	-	-

### 2.3 致病性气道定植菌阳性患者气道定植菌株的分布情况

19例患者中检得气道定植菌22株，其中革兰氏阴性菌19株，占86.36%，革兰氏阳性菌3株，占13.64%。有3例患者检出2种菌株。其中，肺炎克雷伯菌7株（31.83%, 7/22）、铜绿假单胞菌3株（13.64%, 3/22）、流感嗜血杆菌5株（22.73%, 5/22）、大肠埃希氏菌2株（9.09%, 2/22）、阴沟肠杆菌1株（4.54%, 1/22）、表皮葡萄球菌2株（9.09%, 2/22）、金黄色葡萄球菌1株（4.54%, 1/22）、鲍曼不动杆菌1株（4.54%, 1/22）。

### 2.4 两组患者血清SP-D水平的比较

血清SP-D浓度在且在肺癌合并致病性气道定植菌组（31.25±6.09）显著高于非合并组（28.17±5.23）（*P*=0.023）（图 2）。血清SP-D水平与致病性气道定植菌发生呈正相关，相关系数0.204（*P*=0.011）。检出的菌株数量不同患者间血清SP-D水平中位数不同，检出单株致病性定植菌患者血清SP-D水平中位数较检出2株致病性定植菌患者血清SP-D水平明显降低（24.68 *vs* 33.62）（图 3）。

**2:**
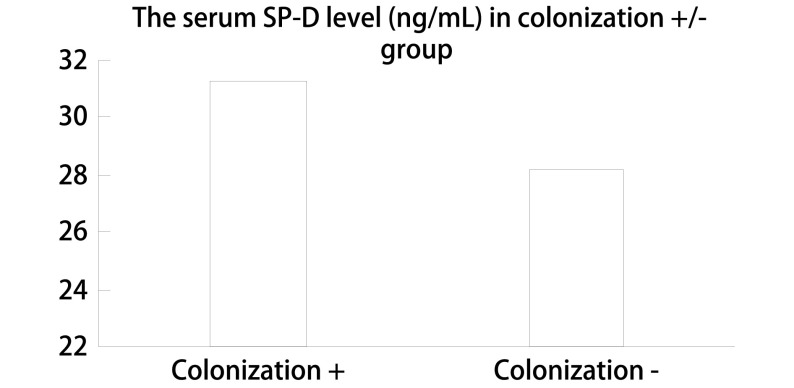
气道定植菌阳/阴性患者血清SP-D水平的比较。血清SP-D浓度在肺癌合并致病性气道定植菌组显著高于非合并组[(31.25±6.09) *vs* (28.17±5.23), *P*=0.023] The comparison of serum SP-D level between positive and negative colonization patients The serum SP-D level of patients in the colonization positive group was higher than that in the colonization negative group [(31.25±6.09) *vs* (28.17±5.23), *P*=0.023]

**3:**
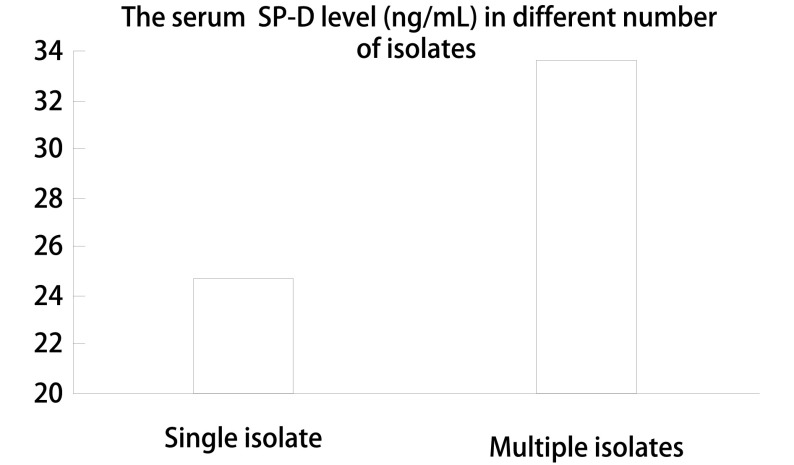
不同气道定植菌菌株数量患者血清SP-D水平中位数的比较。检出单株致病性定植菌患者血清SP-D水平中位数较检出2株致病性定植菌患者血清SP-D水平明显降低（24.68 *vs* 33.62） The median of serum SP-D level in different number of isolates. The serum SP-D level in single isolate group was lower than that in multiple isolates group (24.68 *vs* 33.62)

### 2.5 肺癌合并致病性气道定植菌的高危因素

*Logistic*回归分析结果显示：年龄（>75岁）和吸烟史（吸烟指数≥400支/年）是肺癌患者术前合并致病性定值菌的独立危险因数（表 4）。

**4:** 呼吸道致病性气道定植菌的危险因素的*Logistic*回归 The *Logistic* analysis of the risks of aieway colonizations

Variables in the equation	B	Sig.	95%CI	
			Lower	Upper
Age ( < 75 yr; ≥75 yr)	1.616	0.045	1.039	24.392
Gender	0.025	0.566	0.941	1.118
Smoking index	1.707	0.040	1.080	28.146
Type of pathology	-0.980	0.251	0.071	1.999
Site	1.260	0.216	0.478	26.018
Central lung cancer/Peripheral lung cancer	-0.929	0.372	0.051	3.038
COPD level	23.384	0.999	0.000	-
Diabetes mellitus	0.820	0.578	0.126	40.976
Hypertension	0.981	0.647	0.874	1.980

### 2.6 并发手术后肺炎患者临床病理生理学特征

并发手术后肺炎患者中气道致病性定值菌的发生率是41.67%（5/12），其发生率是无手术后肺炎患者的3.4倍（12.39%; OR=3.363, 95%CI: 1.467-7.711）。并发手术后肺炎的患者在年龄[(64.46±7.31) *vs* (61.78±8.21), *P*=0.021]、吸烟指数≥400支年（83.33% *vs* 52.43%, *P*=0.019）和COPD分级（*P*=0.007）上的差别有统计学意义（表 5）。

**5:** 并发手术后肺炎患者临床病理生理学特征 The clinical demographic characters of patients with POP

	POP (*n*=12)	Non-POP (*n*=113)	*χ*^2^/*t*	*P*
Gender			0.421	0.516
Male	10	85		
Female	2	28		
Age (yr, Mean±SD)	64.46±7.31	61.78±8.21	2.341	0.021
Smoking index (cigarette-year)			5.486	0.019
< 400	2	59		
≥400	10	54		
Type of pathology			2.339	0.310
AC	6	54		
SC	5	58		
Other	1	1		
Resction location			3.291	0.510
LU	3	13		
LL	2	38		
RU	4	31		
RM	1	4		
RL	2	27		
Site of tumor			2.018	0.155
Central lung cancer	8	51		
Peripheral lung cancer	4	62		
COPD level			9.905	0.007
Ⅰ	1	22		
Ⅱ	7	87		
Ⅲ	4	4		
Diabetes mellitus			2.366	0.124
Yes	3	10		
No	9	103		
Hypertension			0.358	0.549
Yes	3	20		
No	9	93		
Colonization			5.578	0.019
Postive	5	14		
Negative	7	99		

## 3 讨论

外科手术仍是目前治疗肺癌的主要手段，肺叶切除及系统纵隔淋巴结清扫是治疗肺癌的主要手术方式。肺癌手术后肺部并发症是影响患者手术后早期恢复的主要原因^[3]^。根据已有的报道，PPC的发生率在2%-40%之间^[9-11]^，常见的肺部并发症包括：术后肺炎、肺不张、急性呼吸衰竭、肺水肿、支气道痉挛以及气胸和持续肺漏气等，术后肺炎是导致患者围术期死亡的主要原因。

近年来，肺癌患者气道致病性细菌定植及其与PPC的关系受到关注^[12, 13]^。致病性细菌定植是指在正常无菌的呼吸道出现了细菌，并可以持续存在且不断生长繁殖，但不引起显著的细菌感染的临床症状^[14]^。细菌定植与感染的区别在于：细菌定植是指细菌在消化道、呼吸道、泌尿生殖道等部位粘膜表面持续存在而未出现宿主反应和不利作用，定植可以是细菌和宿主之间长期持续的共生关系，也可以进一步发展为感染。细菌细菌感染是致病菌或条件致病菌在局部组织或侵入血循环中生长繁殖产生毒素和其他代谢产物所引起急性全身或局部感染，临床上出现相应症状。因此，定植尚不是感染，却是感染的重要来源和最危险的因素。研究^[4]^显示气道致病性细菌定植是PPC的独立危险因素。肺癌患者术前气道致病性细菌定植主要源于：①目前的肺癌病例中，有40%-70%伴发不同程度的COPD^[15]^。研究^[16]^发现，25%-40%的稳定期COPD患者取支气道分泌物进行定量培养发现有气道定植菌存在。②感染因素在肺癌发病机制中有重要的作用，恶性肿瘤同时伴发的免疫抑制和营养状态异常，也为致病性细菌定植提供了条件^[17]^。我们的研究结果显示，大约15%的术前非小细胞肺癌患者存在气道致病性细菌定植。检出的气道定植菌菌株以革兰氏阴性菌为主，其中3例患者检出2种菌株。

通过比较气道定植菌阳性患者与阴性患者的临床资料，我们发现：致病性细菌定植阳性与患者年龄、吸烟史及肿瘤的位置（中央型/周围型）等因素有关，老年免疫力降低，烟雾对于气道功能和微环境的破坏，中央型肺癌导致的气道完全或不完全阻塞是可能的原因。需要指出的是，我们的研究结果提示是否存在呼吸道致病性细菌定植与肺癌患者合并COPD的严重程度无关。虽然，大量COPD患者的致病性细菌定植的研究表明，细菌定植的发生与COPD各项指标有密切相关^[18]^，但目前关于肺癌患者致病性细菌定植的研究很少，已有的文献均未发现^[4]^或没有研究致病性气道定植菌和肺癌手术患者合并COPD相关指标之间的关系^[12, 19]^。我们的研究之所以得出以上的结果，其原因可能是：①对于可以接受手术的肺癌患者而言，即使术前合并不同程度的COPD，其肺功能往往也局限在相对较窄的可以耐受手术的范围内，肺功能较差的患者在术前筛查时就已经被排除；②肺癌的发生本身与致病性细菌定植有交互关系^[17]^，从而削弱了COPD严重程度在肺癌患者致病性细菌定植发生机制上的作用的权重。在进一步的*Logistic*回归分析中我们发现术前发生致病性气道定植菌的危险因素是高龄和长期吸烟史。同时，我们的结果显示：在气道定植菌阳性患者中手术后肺炎的发病率较阴性患者明显增高，合并手术后肺炎的患者中气道致病性定植菌检出率较不发生术后肺炎的患者明显增加，提示肺癌患者术前致病性气道定值菌与手术后肺炎的发生密切相关。

肺表面活性蛋白（surfactant protein, SP）是肺组织局部分泌的重要活性物质，除可以调节表面张力、具有调节蛋白酶-蛋白酶抑制剂之间的平衡状态的功能外，同时还是重要的肺天然免疫分子，可以抵抗病原体侵袭、炎症及氧化应激。已发现的SP有4种，按发现先后命名为SP-A、SP-B、SP-C和SP-D。SP-D是水溶性蛋白，除参与肺泡张力的维持外，还与机体和肺的免疫功能和炎症调节相关。其结构上具有重复表达GIy-X-Y的胶原样区，可与不同细胞表面多种受体结合，参与免疫反应。

SP-D是评价COPD严重程度和预后的重要的生物标志物^[20]^，在COPD的发病和疾病的进展中起到重要作用^[21]^，参与了肺的免疫调节及病原体的吞噬及凋亡机制^[22]^。SP-D由肺泡分泌，肺血气屏障的病变，肺泡细胞间隔增宽，通透性增大，是SP-D入血的主要原因，血清SP-D水平可以反映终末肺组织的器质性变化^[23]^。从理论上设想，局部（支气管肺泡灌洗液中）和血清SP-D的水平高低可以反映术前肺部的免疫状态，并与致病性细菌定植状态相关。我们的研究结果发现致病性定植菌阳性组患者血清SP-D水平较阴性患者增高，血清SP-D水平与致病性气道定植菌发生呈正相关。同时，气道定植菌菌株数量的增加伴随着血清SP-D水平的升高。以上的结果提示肺组织局部免疫功能的变化可能是导致致病性细菌定植的原因之一。

综上所述，在可以手术治疗的肺癌患者中，约占15%的患者伴有并与术前致病性细菌定植，肺癌患者合并致病性气道定植菌与术后肺炎发生密切相关，术前发生致病性气道定植菌的危险因素是高龄和长期吸烟史, 血清SP-D水平与患者术前致病菌定植状态相关。

## References

[1] Islami F, Torre LA, Jemal A (2015). Global trends of lung cancer mortality and smoking prevalence. Transl Lung Cancer Res.

[2] Chen W, Zheng R, Baade PD (2016). Cancer statistics in China, 2015. CA Cancer J Clin.

[3] Stephan F, Boucheseiche S, Hollande J (2000). Pulmonary complications following lung resection: a comprehensive analysis of incidence and possible risk factors. Chest.

[4] Mei J, Liu L, Tang M (2014). Airway bacterial colonization in patients with non-small cell lung cancer and the alterations during the perioperative period. J Thorac Dis.

[5] Kaneda H, Nakano T, Taniguchi Y (2012). Impact of previous gastrectomy on postoperative pneumonia after pulmonary resection in lung cancer patients. Interact Cardiovasc Thorac Surg.

[6] Savardekar A, Gyurmey T, Agarwal R (2013). Incidence, risk factors, and outcome of postoperative pneumonia after microsurgical clipping of ruptured intracranial aneurysms. Surg Neurol Int.

[7] Murphy TF, Brauer AL, Sethi S (2007). Haemophilus haemolyticus: a human respiratory tract commensal to be distinguished from Haemophilus influenzae. J Infect Dis.

[8] Pu Q, Ma L, Che GW (2013). Safety and technical feasibility of single-direction VATS lobectomy: a review of 1, 040 cases. Sichuan Da Xue Xue Bao: Yi Xue Ban.

[9] Murakami J, Ueda K, Hayashi M (2016). Size-capacity mismatch in the lung: a novel predictor for complications after lung cancer surgery. J Surg Res.

[10] Kodra N, Shpata V, Ohri I (2016). Risk factors for postoperative pulmonary complications after abdominal surgery. Open Access Maced J Med Sci.

[11] Lawrence VA, Cornell JE, Smetana GW (2006). Strategies to reduce postoperative pulmonary complications after noncardiothoracic surgery: systematic review for the American college of physicians. Ann Intern Med.

[12] Belda J, Cavalcanti M, Ferrer M (2005). Bronchial colonization and postoperative respiratory infections in patients undergoing lung cancer surgery. Chest.

[13] Hsu-Kim C, Hoag JB, Cheng GS (2013). The microbiology of postobstructive pneumonia in lung cancer patients. J Bronchology Interv Pulmonol.

[14] Yang XH, Wang HY (2011). Relation of change of COPD lower aieway bacterial colonization with lung function and sptum cytokines. Zhongguo Quan Ke Yi Xue.

[15] Loganathan RS, Stover DE, Shi W (2006). Prevalence of COPD in women compared to men around the time of diagnosis of primary lung cancer. Chest.

[16] Moghaddam SJ, Ochoa CE, Sethi S (2011). Nontypeable haemophilus influenzae in chronic obstructive pulmonary disease and lung cancer. Int J Chron Obstruct Pulmon Dis.

[17] Moghaddam SJ, Barta P, Mirabolfathinejad SG (2009). Curcumin inhibits COPD-like airway inflammation and lung cancer progression in mice. Carcinogenesis.

[18] Sethi S, Maloney J, Grove L (2006). Airway inflammation and bronchial bacterial colonization in chronic obstructive pulmonary disease. Am J Respir Crit Care Med.

[19] Oor JE, Daniels JM, Debets-Ossenkopp YJ (2016). Bronchial colonization and complications after lung cancer surgery. Langenbecks Arch Surg.

[20] Sin DD LR, Gan WQ, Man SP (2007). Circulating surfactant protein D as a potential lung-specific biomarker of health outcomes in COPD: a pilot study. BMC Pulm Med.

[21] Lomas DA, Silverman EK, Edwards LD (2009). Serum surfactant protein D is steroid sensitive and associated with exacerbations of COPD. Eur Respir J.

[22] Ofek I, Mesika A, Kalina M (2001). Surfactant protein D enhances phagocytosis and killing of unencapsulated phase variants of Klebsiella pneumoniae. Infect Immun.

[23] Carlson TK, Torrelles JB, Smith K (2009). Critical role of amino acid position 343 of surfactant protein-D in the selective binding of glycolipids from *Mycobacterium tuberculosis*. Glycobiology.

